# Glycogen synthase kinase 3ß participates in late stages of Dengue virus-2 infection

**DOI:** 10.1590/0074-02760190357

**Published:** 2020-02-27

**Authors:** Alexandra Milena Cuartas-López, Juan Carlos Gallego-Gómez

**Affiliations:** 1 Universidad de Antioquia, Institute of Medical Research, School of Medicine, Group of Molecular and Translational Medicine, Medellín, Colombia

**Keywords:** GSK3ß, Dengue virus, cell signaling, PI3K/Akt, viral infection

## Abstract

**BACKGROUND:**

Viruses can modulate intracellular signalling pathways to complete their infectious cycle. Among these, the PI3K/Akt pathway allows prolonged survival of infected cells that favours viral replication. GSK3β, a protein kinase downstream of PI3K/Akt, gets inactivated upon activation of the PI3K/Akt pathway, and its association with viral infections has been recently established. In this study, the role of GSK3β during Dengue virus-2 (DENV-2) infection was investigated.

**METHODS:**

GSK3β participation in the DENV-2 replication process was evaluated with pharmacological and genetic inhibition during early [0-12 h post-infection (hpi)], late (12-24 hpi), and 24 hpi in Huh7 and Vero cells. We assessed the viral and cellular processes by calculating the viral titre in the supernatants, In-Cell Western, western blotting and fluorescence microscopy.

**RESULTS:**

Phosphorylation of GSK3β-Ser9 was observed at the early stages of infection; neither did treatment with small molecule inhibitors nor pre-treatment prior to viral infection of GSK3β reduce viral titres of the supernatant at these time points. However, a decrease in viral titres was observed in cells infected and treated with the inhibitors much later during viral infection. Consistently, the infected cells at this stage displayed plasma membrane damage. Nonetheless, these effects were not elicited with the use of genetic inhibitors of GSK3β.

**CONCLUSIONS:**

The results suggest that GSK3β participates at the late stages of the DENV replication cycle, where viral activation may promote apoptosis and release of viral particles.

The glycogen synthase kinase-3 (GSK-3) is a multifunctional monomeric protein that participates as an intermediary in several signalling pathways, including the Wnt/β-catenin, Hedgehog and PI3K/Akt pathways. Several mechanisms and molecules can activate GSK-3, including activation of cytokine receptor, heterotrimeric G protein-coupled receptors and tyrosine kinase receptors. The role of GSK was identified in the metabolism of glucose through phosphorylation and subsequent inhibition of the glycogen synthetase enzyme and insulin signalling. However, GSK-3 was later identified as a protein having serine-threonine kinase activity that regulates different cellular processes such as gene transcription, embryonic development, translation, cytoskeletal organisation, cell cycle progression and regulation of pro and anti-apoptotic pathways. Therefore, GSK-3 activity is tightly modulated by cells.^1^

GSK3 is highly conserved and plants, fungi, flies, helminths, and vertebrates exhibit orthologous proteins. In mammals, there are two homologous forms of GSK3 gene product: GSK3α of 51 kDa (located on chromosome 19) and GSK3β of 47 kDa (located on chromosome 3), which possess 85% similar and 98% homologous sequences within their kinase domains.^2^ However, these proteins are not functionally homologous or redundant. GSK3β, better studied, is constitutively active in resting cells and is inhibited upon activation of any signalling pathways in which it is involved.^1^ This kinase is mainly found in cytoplasm and nucleus, but it can also be found in mitochondria where its activity is regulated. Regulation of GSK3β by phosphorylation has been extensively studied. Phosphorylation at serine 9 (Ser9) and tyrosine 216 (Tyr216) lead to GSK3β inactivation and activation, respectively. In addition, formation of protein complexes, intracellular localisation, and certain stabilising drugs influence GSK3β modulation.^3^

Impairment of GSK3β function have been described in several disorders and diseases including cancer, cardiovascular disease and neurological disorders such as Alzheimer’s disease, bipolar disorders, and Huntington’s disease. GSK3β is also involved in neoplastic transformation and development of hepatocellular cancer.^4^

A few investigations have described participation of GSK3β in inducing apoptosis in viral infections including those caused by varicella-zoster virus (VZV), hepatitis C virus (HCV), human immunodeficiency virus-1 (HIV-1), Venezuelan equine encephalitis virus (VEEV), coxsackievirus and enterovirus 71 (EV71).^5^ ^,^ ^6^ ^,^ ^7^ ^,^ ^8^

In infections caused by Dengue virus (DENV), GSK3β regulates transcription factor NF-κB, leading to production of nitric oxide (NO) and induction of apoptosis. This signal is triggered by binding of DENV anti-NS1 antibodies to cells.^9^ DENV-2 inhibits GSK-3 activity to induce expression of MHC Class-1-related chain (MIC) A and MIC-B, and IL-12 production in monocyte-derived dendritic cells (Mo-DCs).^10^

Considering that PI3K/Akt kinase pathway is involved in the infection of epithelial cells, Huh-7 and Vero, by DENV-2^11^ and that GSK3β is downstream of this cascade, it is intriguing to evaluate the role of GSK3β in the infective process of DENV-2. Current reports on the participation of GSK3β activity in DENV-2 infection process has been contrasting. In some settings, GSK3β activation leads to apoptosis, while in other conditions it seemed to induce cell proliferation.^12^ Furthermore, GSK3β pathway has been hypothetically postulated as crucial in modulating Drosha microprocessor activity and microRNA biogenesis that could be the trigger of important events involving microRNAs at early stages of DENV infection.^13^ Likewise, several families of miRNAs including miR-34, miR-15, and miR-517 families have been reported to inhibit multiple flaviviruses such as DENV, West Nile virus (WNV) and Japanese encephalitis virus. Members of miR-34 family can repress Wnt/β-catenin signalling with antiflaviviral effects, modulating type I interferon (IFN) signalling pathways by binding of GSK3β to TANK-binding kinase 1(TBK1).^14^

In this work, the role of the protein GSK3β during Dengue virus infection was investigated in Huh7 and Vero cells. Importantly, we compared GSK3β activation during the stages of infection and assessed its influence on cellular responses and viral release.

## MATERIALS AND METHODS

*Cell culture* - Viruses were cultured in C6/36 HT (high temperature) cells from *Aedes albopictus*. Virus cultures were titrated in Vero cells (from African green monkey kidney, ATCC number CCL-81); these cells and Huh7 cells (human hepatoblastoma, donated by Dr Priscilla Yang, Harvard Medical School, Boston, MA, USA) were used for evaluation assays of GSK3β pathway. Specific monoclonal antibodies against DENV E protein (αE) were obtained from culture supernatants of 4G2 hybridoma cells (ATCC number: HB-112). Vero, Huh7, and C6/36 HT were maintained in Dulbecco’s Modified Eagle Medium (DMEM) (Gibco) supplemented with 1-10% FBS (Gibco); 4G2 cells were grown in Hybry-care medium (ATCC), all supplemented with 1% penicillin/streptomycin (Sigma-Aldrich, St. Louis, MO). All cells were maintained in 5% CO_2_ atmosphere at 37ºC, except for C6/36 HT, which was maintained at 34ºC.

*Pharmacological inhibitors and antibodies* - GSK3β small molecule inhibitor Kin-001-184 was donated by Dr Priscilla Yang (Harvard Medical School). CT 99021 (Kin-001-157) was obtained from Axon (cat # 1386 Groningen - The Netherlands). Mycophenolic acid (MPA), obtained from Sigma-Aldrich (Ref. M3536-250G), was used as positive control for the inhibition of DENV replication. GSK3β inhibitors were dissolved in dimethyl sulfoxide (DMSO, Sigma) and MPA was dissolved in methanol (50 mg/mL). The primary antibodies used were rabbit α-GSK3β (cat # 9369), rabbit α-phospho-GSK3β-Ser9 (cat # 9323), rabbit α-Akt (cat # 9272), rabbit α-phospho-Akt-Ser473 (cat # 9271S), rabbit α-GADPH (cat # 2118), and rabbit α-β-catenin (cat # 9587) (Cell Signalling, Danvers, MA). For immunofluorescence, secondary antibodies conjugated to fluorophores Alexa 488 and Alexa 594 (Molecular Probes, Eugene, OR) were used, and Hoechst 33258 (Thermo Fisher Scientific, cat # H3569) was used for nuclear labelling. The secondary antibodies used were IRDye 800CW goat anti-mouse and IRDye 680 goat anti-rabbit (1:15000) (Li-COR, Lincoln, NE). Protein quantification was performed using BCA Protein Assay kit (Pierce, Thermo Scientific ref 23225).

*Cytotoxicity assay* - Following treatments with inhibitors, the viability of Huh7 cells was tested using the MTT (3- (4,5-Dimethylthiazol-2-yl) -2,5-Diphenyltetrazolium bromide) assay. Cells were seeded onto 96-well plates and incubated for 24 h. The culture medium was replaced with DMEM-containing GSK3β or MPA inhibitors at concentrations of 5, 10, 20, and 40 μM, prepared by serial dilution. After 24 h incubation, the medium was replaced with 50 μL of MTT [0.5 mg/mL in phosphate-buffered saline (PBS)], followed by 3 h of incubation at 37ºC. DMSO (100 μL) was added to solubilise formazan crystals and incubated for 15 min. Absorbance at 450 nm measured using a microplate reader (Benchmark, Bio-Rad Laboratories, Hercules, CA, USA). Three independent experiments were performed with each treatment in triplicates.

*Virus growth and titration* - The prototype strain DENV-2 New Guinea C (NGC) donated by Maria Elena Peñaranda and Eva Harris (Sustainable Sciences Institute and the University of California) was used in all infection experiments. Virus stocks were used for infection of C6/36 HT cells at low multiplicity of infection (MOI) (0.01 PFU/cell). Once infected, cells were incubated for seven days and supernatants were aliquoted and stored at -80ºC until titration. Viral titre determination was performed by diluting virus (10^-1^-10^-5^) in serum-free medium. Vero cell monolayers grown to 90% confluence in 48-well plates were inoculated with diluted virus. After 1 h adsorption at 37ºC, viral inoculum was removed. Cells were washed with PBS and covered with 2% carboxymethyl cellulose (medium viscosity carboxymethyl cellulose, Sigma-Aldrich) in DMEM containing 2% foetal bovine serum (FBS). After seven days of incubation, cells were fixed with 4% paraformaldehyde and stained with 0.5% violet crystal prepared in 20% methanol. Viral titre calculations were done by counting two replica plates from three independent experiments (n = 6).

*Flow cytometry of infected cells* - Huh7 cells (2 × 10^5^) were seeded onto 6-well plates for 24 h. The cells were washed once with warm trypsin-supplemented PBS and twice with PBS. Cells were resuspended in 500 μL of PBS and labelled with DIOC6 (to measure the mitochondrial membrane potential) and propidium iodide (PI3-A, to assess cell membrane damage).

*Assessment of GSK3β phosphorylation using In-Cell Western* - Activation kinetics of GSK3β was done *in situ* using In-Cell Western. Briefly, 2.5 X 10^4^ Huh7 cells were seeded into each well of 96-well plates and incubated in 2% FBS-containing medium. To cease activation of signalling pathways by growth factors, culture medium was replaced with serum-free medium 24 h later, followed by 2 h of incubation at 37°C. The medium was subsequently removed and the wells were washed once with warm preheated PBS. Cells were infected with DENV-2 at a MOI of 5 in a final volume of 25 μL/well for indicated times (1 min to 2 h); cells were washed with cold PBS, fixed with 4% paraformaldehyde (PFA), and incubated at room temperature for 20 min with gentle agitation. After five 5-min washes with wash solution (Triton 0.1% in PBS) with gentle agitation, cells were incubated with 150 μLof blocking solution (LICOR ODYSSEY blocking buffer) and incubated for 90 min at room temperature under moderate agitation. Subsequently, the blocking solution was removed and cells were incubated for 2 h at room temperature with either rabbit α-pGSK3β-Ser-9 (1:100) or mouse α-GSK3β (1:100). After thorough washes, cells were incubated secondary antibodies IRDye goat α-rabbit 800D or IRDye 680RD goat α-mouse diluted 1: 500 (diluted in ODYSSEY LICOR blocking buffer) at room temperature for 1 h with gentle agitation. Cells were then washed thoroughly with wash solution. All wash solution were completely removed from wells and cells were analysed using Odyssey Infrared Imaging System, software version 3.0 (Li-COR). α-pGSK3β-Ser-9 values were normalised to baseline GSK3β protein levels.

*Evaluation of GSK3β small molecule inhibitors* - Two small molecule inhibitors of GSK3β were evaluated in Huh7 cells prior to or following infection with DENV-2 for three different time points (0-24 h) and the more effective inhibitor was chosen for further experiments. (1) Pre-infection treatment: 3 h before infection, cells were treated with Kin-001-157 inhibitor (iGSK3β) at concentrations of 20 or 40 μM. Prior to infection, medium was removed and cells washed with pre-warmed PBS. Viral inoculum was added (DENV-2 MOI = 10) and infection maintained for 1 h. Cells were washed and subsequently incubated in 2% FBS, drug-free DMEM for 24 h. (2) Early infection treatment [0-12 h post-infection (hpi)]: cells were infected with DENV-2 (MOI = 10) diluted in DMEM containing iGSK3β, and incubated for 1 h. Cells were washed and medium replaced with 2% FBS-DMEM containing inhibitor and incubated for 11 h. Medium was replaced using inhibitor-free, 2% FBS medium and cells were incubated for 12 h. (3) Late infection treatment (12-24 hpi): cells were infected with DENV-2 (MOI = 10) diluted in serum-free medium and incubated for 1 h. Cells were washed with PBS and incubated for 11 h in 2% DMEM Medium was replaced with 2% FBS-DMEM containing iGSK3β and cells were incubated for 12 h. The concentrations of iGSK3β tested were 20 and 40 μM. Supernatants were collected after a total of 24 h post-infection or treatment and monolayers fixed with 4% PFA for immunofluorescence assays or lysed for western blotting.

*GSK3β silencing with siRNA and shRNA* - Silencing of GSK3β was carried out using two methodologies: cells were transfected with plasmids with gene sequences expressing short hairpin RNAs (shRNAs) or a commercial pool of small interfering RNAs (siRNAs).

*Reverse transfection of shRNAs* - Three different versions of lentiviral vector pCMV-GIN-ZEO-GSK3β expressing green fluorescent protein (GFP) were used.^15^ Versions 1 and 3 (Ver-1 and Ver-3) express shRNAs targeting GSK3β and have been previously validated,^16^ and the scrambled version (Ver-2) contained nontargeting specific sequences. Briefly, 4.0 μg of lentiviral DNA (quantified using the Nanodrop system) was dissolved in 500 μL of Opti-DMEM medium (serum-free medium in 6-well plates). Four μL of Lipofectamine (Invitrogen) was added to the DNA, gently mixed, and incubated for 20 min at room temperature. Huh7 or Vero cells suspension (in 2% FBS-DMEM, 2 x 10^5^ cells/well) was added into the DNA/Lipofectamine mixture in the wells, and incubated at 37ºC. After 48 h of incubation, the transfection efficiency was confirmed by GFP expression for fluorescence using the TYPHOON 9400 imager. Cells expressing ≥ 50% GFP efficiency were infected 48 h post-transfection (hpi). The supernatant was removed from cells 24 hpi (72 hpi) before lysing. Cell lysates were stored at -70ºC until analysis.

*Reverse transfection of siRNAs* - A pool of six different siRNAs directed against GSK3β was used. For the negative control, nontargeting siRNA (NT Pool) was used. For transfection, 6 pmol siRNA/well was dissolved in 200 μL Opti-MEM in 12-well plates and mixed gently; 1 μL of Lipofectamine RNAiMAX was added to each well, mixed, and incubated at room temperature for 20 min. Huh7 or Vero cell suspension (in 2% FBS-DMEM, 2 × 105 cells/well) was added to the DNA/Lipofectamine mixture in wells and incubated for 24 h at 37ºC, prior to infection, for the indicated times. The supernatant was removed from cells 24 hpi (72 hpi) before lysing. Cell lysates were stored at -70ºC until analysis.

*Western blotting* - Cells were lysed with lysis buffer (150 mM NaCl, 20 mM Tris pH 7.4, 10% glycerol, 1 mM EDTA, 1% NP40, and 1 mg/mL protease inhibitor cocktail). Twenty μg of protein in the loading buffer (0.375 M Tris, pH 6.8, 50% glycerol, 10% SDS, 0.5 M DTT, and 0.002% bromophenol blue) was denatured by heating at 100ºC for 5 min before gel electrophoresis [10% sodium dodecyl sulphate-polyacrylamide gel electrophoresis polyacrylamide gels (SDS-PAGE)] using a Mini-Protein System (Bio-Rad). Separated proteins were transferred onto nitrocellulose membranes (Amersham, GE, Boston, MA) in a Mini Trans-Blot electrophoretic transfer cell at 250 mA for 2 h. Membranes were washed using wash buffer, T-TBS (20 mM Tris-HCl pH 7.5, 500 mM NaCl, 0.05% Tween-20 in buffered saline, pH 7.4), and blocked with 5% of skimmed milk for 1 h. Membranes were incubated overnight at 4ºC with the appropriate primary antibodies: Rabbit α-pAkt Ser-473 (1:500), rabbit α-p-GSK3β-Ser9 (1:500), undiluted 4G2 α-Envelope antibody (α-ENV), or mouse α-GADPH (1:1000). Membranes were thoroughly washed and incubated with peroxidase-coupled anti-rabbit or anti-mouse secondary antibodies (1: 5000, Pierce). Signals were developed using electrochemiluminescence (ECL, Thermo Scientific) and imaged with autoradiographic films (Hyperfilm ECL, Amersham or AGFA RP2 plus films).

*Fluorescence microscopy* - Huh7 cells were prepared for fluorescence microscopy according to Cuartas et al.^11^ Briefly, cells were seeded on coverslips in 24-well plates at a density of 5 x 10^4^ cells per well. At 24 hpi, cells were washed with cytoskeleton buffer (CB) and fixed with 3.8% PFA at 37ºC for 30 min. Cells were permeabilised with 0.5% Triton X-100 in CB. Cells were blocked with 5% FBS in CB and subsequently incubated with undiluted primary αE antibody. After thorough washes, cells were simultaneously incubated with anti-mouse secondary antibody conjugated to Alexa 594, phalloidin Alexa 488 (for actin labelling) and Hoechst 33258 (for core labelling, 1: 5000) followed by washes with CB. Fluorescence were evaluated using an epifluorescence microscope (IX-81 Olympus), and images captured by software (Media Cybernetics, Image-Pro Plus). Confocal imaging was obtained using a FluoView FV1000 Confocal Microscope (Olympus).

*Image analysis* - Quantification of RGB images obtained by fluorescence microscopy was performed in Fiji (Distribution of ImageJ 2.0.0.). For contrast enhancing of images (gray value histogram-based approach), pixels saturated at 0% were used to define intensity thresholds. Measurements of integrated density and mean of area gray values for each cell and its background were used to estimate fluorescence response of DENV E protein in cells. The DENV E protein fluorescence response is defined as the mean intensity of the gray values assigned to every pixel within a defined cell area whose value is higher than the intensity of the background pixels.

*Statistical analysis* - Analysis of variance (ANOVA) was performed. Error bars correspond to 95% confidence interval. Analyses were carried out using PRISM 8 statistical package. Results were considered significant if type II statistical error was 95%.

## RESULTS

**Fig. 1:**
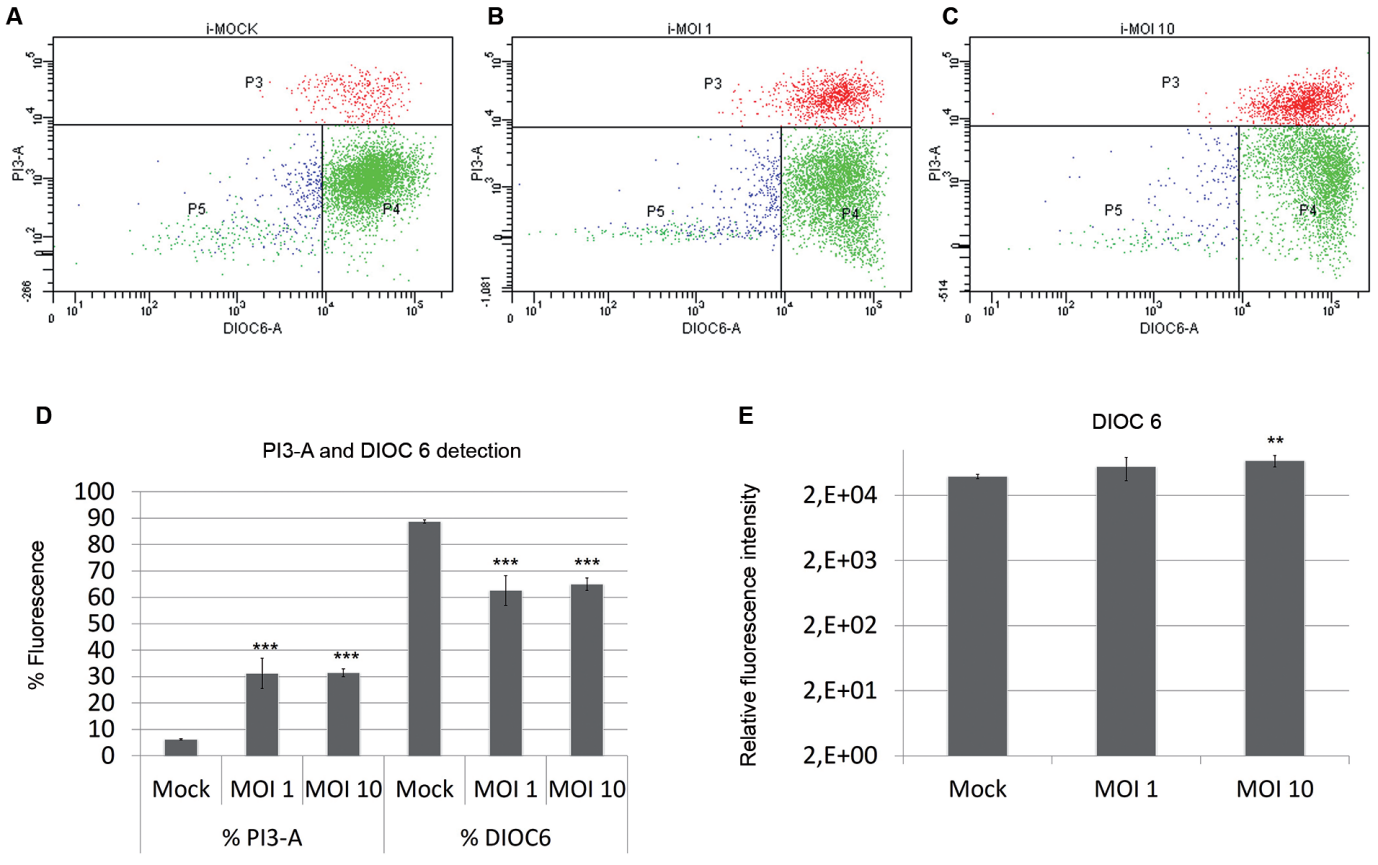
Dengue virus-2 (DENV-2) causes damage to cell flow cytometry of uninfected cells (MOCK) (A), Huh7 cells infected at multiplicity of infection (MOI) 1, (B) and MOI 10 (C). Percent fluorescence of PI3-A and DIOC6 indicated beginning of cell death (D). Fluorescence intensity of DIOC6 increased in DENV-2-infected cells compared to uninfected cells (E). Results are presented as mean ± standard deviation (SD) (n = 3 independent experiments).

*Infection of Huh7 cells with DENV-2 caused damage to cell membranes* - Effect of DENV-2 infection on cell membrane and mitochondria activity was tested in Huh7 cells. Damage to cell membrane following infection with DENV-2 at different MOIs was assessed by flow cytometry measurement of PI3-A. Levels of PI3-A increased in cells infected at MOI 1 and 10, compared to uninfected cells (Fig. 1A-C). A decrease in mitochondrial activity of infected cells was noted at both MOI (Fig. 1D). However, at MOI of 10, fluorescence intensity of DIOC6 in infected cells increased (Fig. 1E).

**Fig. 2:**
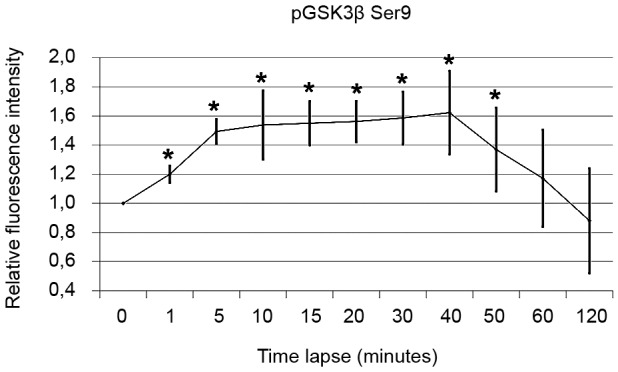
Dengue virus-2 (DENV-2) induces inhibitory phosphorylation of GSK3β (Ser9) in Huh7 cells relative fluorescence intensity showing the inhibitory phosphorylation of GSK3β at Ser9 detected by In-Cell Western blotting in DENV-2-infected cells compared with uninfected cells (0 min). (Values of pGSK3beta Ser9 normalised to baseline GSK3beta). Results are presented as mean ± standard deviation (SD) (n = 3 independent experiments).

*DENV-2 induces inhibitory phosphorylation of GSK3β (Ser9) in Huh7 cells* - To assess changes to GSK3β activities during infection with DENV-2, we performed dose dependent infection experiments for up to 2 h and evaluated GSK3β phosphorylation status *in situ* using In-Cell Western. An inhibitory phosphorylation of GSK3β-Ser9 was observed in Huh7 cells after 1 min of infection with DENV-2. p-GSK3β remained sustained through 50 min post-infection (Fig. 2). This suggests that GSK3β becomes inactivated very early during DENV-2 infection.

**Fig. 3:**
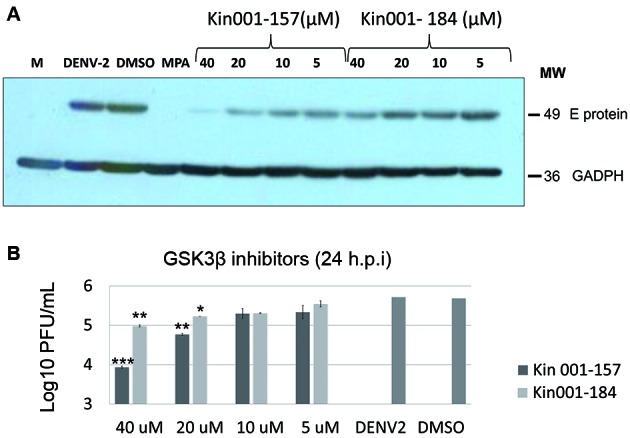
treatment using small molecule inhibitors of GSK3β affects the infection with Dengue virus-2 (DENV-2) when cells are treated over the course of the infection. (A) Western blot demonstrating effect of two GSK3β small inhibitory molecules on detection of intracellular DENV E protein is observed showing protein reduction in a dose-dependent manner by using Kin-001-157 and Kin-001-184. (B) Titration of supernatants from infected cells treated with Kin-001-157 and Kin-001-184; dose-dependent decrease in viral titres was observed with both inhibitors, with greater decrease in Kin 001-157-treated cells. Results are presented as mean ± standard deviation (SD) (n = 3 independent experiments).

*Continuous inhibition of GSK3β modulated DENV-2 activities* - The effect of two small molecule inhibitors of GSK3β on DENV-2 infection was assessed. According to previous reports, Kin-001-184 and Kin-001-157 inhibit GSK3β with high specificity.^17^ A decrease in the intracellular DENV E protein was detected when Vero and Huh7 cells were treated with non-cytotoxic concentrations (5, 10, 20, and 40 μM) of inhibitors over infection period (0-24 hpi), compared to untreated infected cells (Fig. 3A). Culture supernatant exhibit dose-dependent reduction in viral titre following continuous treatment of cells with Kin-001-157 at 20 and 40 μM resulted in 0.9- and 1.8-log reduction in viral titre was observed, respectively. However, treatment with Kin-001-184 resulted in only 0.7 and 0.5 Log reduction in viral titre at 40 and 20 μM concentrations, respectively (Fig. 3B). Consequently, Kin-001-157 was chosen for subsequent experiments given its better inhibitory actions on viral activities.

**Fig. 4:**
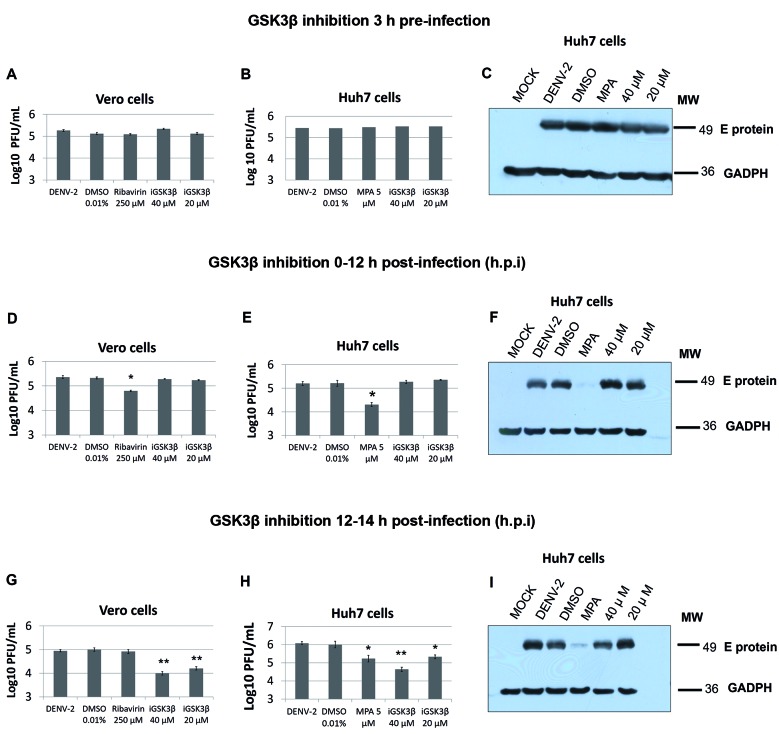
GSK3β inhibitor Kin-001-157 selectively affects late but not early stages of Dengue virus-2 (DENV-2) infection viral titres at 24 h post-infection (hpi) in Vero and Huh7 cells 3 h pre-infection (A, B); early (D, E) or late (G, H) treatment with iGSK3. Reduction of viral titres in cells treated for 12-24 hpi (C, F, I). Corresponding levels of DENV-2 E in Huh7 cells, as detected by western blot. No reduction in DENV E was observed in both the treatments. Plaque assay results are presented as mean ± standard deviation (SD). Results are representative of three independent experiments. *p < 0.1; **p < 0.05.

*Inhibition of GSK3β selectively affected the late but not the early stages of DENV-2 infection* - Since viral infection is a multi-stage process, we employed three strategies to delineate the role of GSK3β at infection stages in the Vero and Huh7 cells: pre-infection treatment (3 h prior) at the early (0-12 hpi) and late post-infection timepoints (12-24 hpi). Inhibiting GSK3β did not affect viral titres or the amount of intracellular DENV E protein in pre-treated cells (Fig. 4A-C) or at early infection (0-12 hpi) (Fig. 4D-F). In contrast, treatment of cells with iGSK3β during the later stages of infection (12-24 hpi) resulted in a reduction of viral titres. In Vero cells, 1.1-log reduction in the viral titre at inhibitor concentrations of 40 and 20 μM (Fig. 4G) was observed. In Huh7 cells, viral titres decreased by 1.4 and 0.8 Log at 40 μM and 20 μM, respectively (Fig. 4H); there were no changes in the DENV E levels as detected by western blotting (Fig. 4I). Mycophenolic acid (MPA) and ribavirin treatment were used as positive controls due to their demonstrated inhibitory effects on DENV virus replication.

**Fig. 5:**
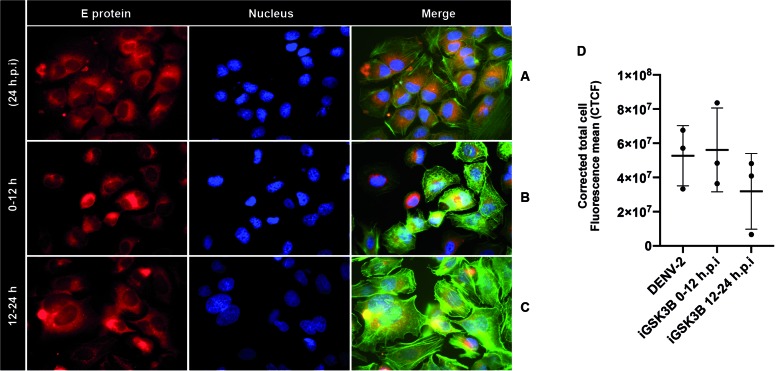
subcellular distribution of the viral envelope protein is not affected by the inhibitory treatment of GSK3β in Huh7 cells infected with Dengue virus-2 (DENV-2). (A) Untreated infected cells, where a homogeneous cellular distribution of DENV E protein is observed. Cells infected and treated 0-12 hpi (B) and 12-24 hpi (C); no significant changes were observed in DENV-2 E levels. (D) The inhibition of GSK3b does not affect DENV envelope protein synthesis. Comparison and differences of corrected total cell fluorescence mean (CTCF) for the treatment GSK3β at different times post-infection (hpi). Infection Control (DENV-2). p values for paired sample comparison were determined using one-way ANOVA. Error bars, 95% confidence interval (CI).

*Subcellular distribution of viral envelope protein remained unaffected by GSK3β inhibition in DENV-2-infected Huh7 cells* - We investigated the distribution of viral proteins at various time points following infection with DENV-2 in the presence or absence of iGSK3β in Huh7 cells. A homogeneous intracellular distribution pattern was observed for DENV E in infected, treated or untreated Huh7 cells (Fig. 5A). Neither treatment with GSK3β inhibitor at early infection stages 0-12 hpi (Fig. 5B) nor at late infection stages 12-24 hpi (Fig. 5C), had significant effects on DENV E distribution pattern. Statistical analysis indicated no significant change (Fig. D) in fluorescent intensity of DENV E protein as infection control and GSK3β treatments were compared (p = 0.3898).

**Fig. 6:**
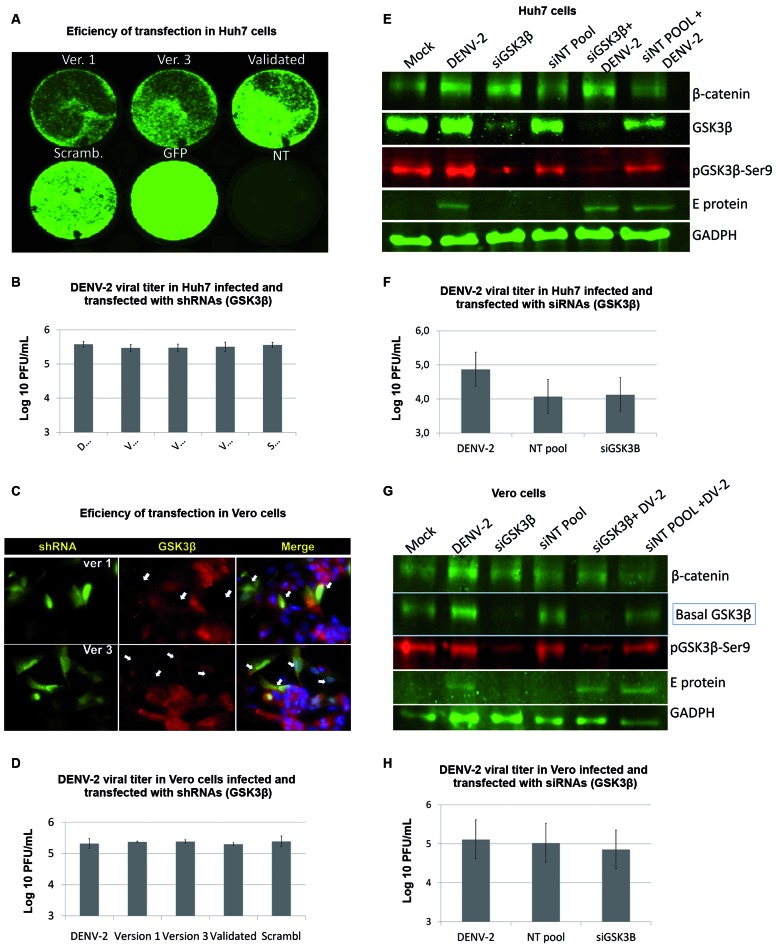
GSK3β knockdown in Vero and Huh7 cells do not affect Dengue virus-2 (DENV-2) infection. Transfection efficiency in Huh7 (A) and Vero cells (E) using validated plasmid shRNAs for GSK3β, Version 1 (Ver.1), version 3 (Ver.3), or scrambled, assessed for GFP at 48 hpi (B, F) Viral titre corresponding to 72 hpi*.* and 24 hpi western blot of Huh7 (B) and Vero cells (G) transfected with siRNAs demonstrating GSK3β silencing. Viral titres of infected Huh7 (D) and Vero cells (H) treated with siRNAs for GSK3β. Plaque assay results were presented as mean ± standard deviation (SD). Results are representative of three independent experiments.

*Knockdown of GSK3β had no effect on DENV-2 infection of Vero and Huh7 Cells* - Loss-of-function experiments were carried out in Vero and Huh7 cells using shRNAs and siRNAs that targeted GSK3β. Silencing of GSK3β was evaluated by GFP protein expression from vectors bearing interfering shRNAs pCMV-GIN-ZEO-GSK3β (Ver1 and Ver3), which were detected 48 hpi using TYPHOON 9400 scanner for Huh7 cells (Fig. 6A), and fluorescence microscopy for Vero cells (Fig. 6C). The amount of GSK3β protein in cells expressing GFP at baseline (arrowheads, Fig. 6E) was also tested. An expression efficiency was observed at 48 h, the time point at which transfected cells were infected. Supernatants of infected and knocked-down cells were subsequently titrated 24 hpi and 72 hpi (Fig. 6B, D). In addition, potent knockdown of GSK3β with siRNAs in Huh7 and Vero cells was confirmed by western blotting using infrared detection of Odyssey System (Fig. 6E, G). However, no decrease in viral titres was observed in both Vero and Huh7 cells (Fig. 6F, H).

**Fig. 7:**
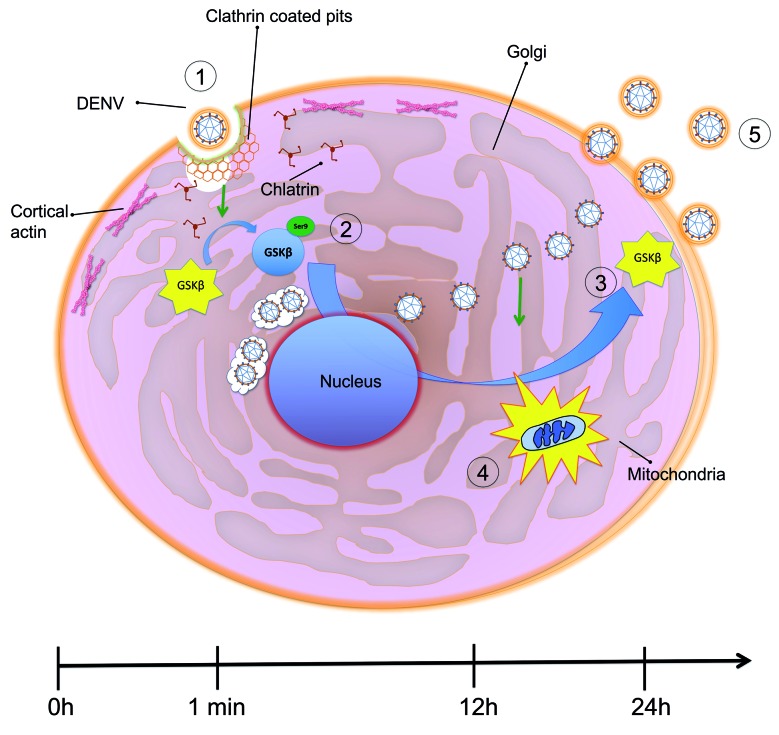
model for GSK3β modulation during Dengue virus-2 (DENV-2) infection. (1) The infection with DENV-2 induces inactivation of GSK3β signalling (2) with serine 9 phosphorylation, shortly after infection. (3) GSK3β inactivation during late infection (12-24 hpi) affects mitochondrial function (4), favouring DENV-2 release from the infected cells (5).

Fig. 7 is a schematic of the main findings of the role of GSK3β in the infection by Dengue virus.

## DISCUSSION

The involvement of PI3K/Akt signalling pathway proteins including GSK3β and several other downstream effectors in viral infections has been described. GSK3β participates in the infection cycle of some viruses such as enterovirus,^18^ human papillomavirus (HPV),^19^ varicella-zoster virus (VZV),^5^ hepatitis C virus (HCV),^8^ ^,^ ^20^ among other. PI3K/Akt signalling pathway is activated during infection cycle resulting in apoptosis.^21^ ^,^ ^22^ ^,^ ^23^ ^,^ ^24^ Nonetheless, the role of GSK3β in this process is not fully understood.

We previously reported that DENV-2 infection causes activation of Akt in Huh7 and Vero cells.^11^ Activation of Akt pathway during the infection with DENV and the Japanese encephalitis virus is associated with apoptosis inhibition.^21^ However, proteins in signalling pathway downstream of active DENV infections remain unidentified. Activation of Akt and downstream inactivation of GSK3β inhibit cell death and modulate cell cycle regulation by cyclin-D1,^25^ implicating inactivation of GSK3β as potential requirement for the inhibition of extrinsic pathway-triggered apoptosis during early viral infection. This hypothesis is consistent with findings from the current study in which we observed Ser9 phosphorylation and inactivation of GSK3β at early-infection time points. The inactivation of GSK3β would explain the lack of effect on virus production upon chemical inhibition of GSK3β at this stage of infection.

We did not investigate activation of PI3K/Akt and downstream inactivation of GSK3β using UV-inactivated viruses, as our focus was on delineating the specific role of DENV-2 infection with active virions. However, Hilde M van der Schaar et al.^26^ suggested that activation of Akt pathway occurs upon engagement of cell receptors by the virus. The study presented DENV tracking in living cells, where authors detected that single DENV particles are able to bind membrane regions enriched with clathrin-coated pits only 48 s after infection. Whereas at 94 seconds, the clathrin signal rapidly disappears indicating disassembling of the clathrin shell required for the subsequent internalisation of the vesicle. Fusion of viral membrane with late endosomes occurred 512 s post-infection. Based on this work and our findings, we presume that activation of PI3K/Akt pathway and subsequent phosphorylation of GSK3β as early as 1 min post-infection occur upon virus binding to the cell receptor involved in activation of the pathway even before viral endocytosis begins.

Our findings on the treatment of infected cells with iGSK3β later in the replication cycle (12-24 hpi) were also consistent with what is expected on apoptosis induction during viral infections for the release of new enveloped viral particles of viruses such as DENV. If GSK3β plays a role in the induction of cell death during DENV-2 infection, a late inhibition would affect mitochondria-dependent apoptosis, which can be regulated by GSK3β,^27^ and thus influence viral release or intracellular trafficking of viral particles. Our flow cytometry experiment data suggest that this phenomena may occur by means of mitochondrial intrinsic apoptosis, considering a statistically significant reduction in mitochondrial activity (DIOC6) in cells infected at MOI = 1 and MOI = 10, compared with uninfected cells.

Although results obtained from small molecule inhibitors of GSK3β and interfering RNAs (shRNAs and siRNAs) did not show similar results related to a decreased viral infection, the lack of a GSK3β silencing effect on the infection could likely be explained by the activity of non-silenced protein. Although the use of interfering RNAs (siRNAs) resulted in a remarkable decrease in the amount of GSK3β, as seen via western blotting, this reduced protein level does not affect normal functioning during DENV-2 infection (12-24 hpi). Similar results on the efficacy of pharmacological inhibitors, compared to genetic inhibitors, have been observed in studies involving other viruses.^28^ In our study, this might be explained by limited silencing of a single protein isoform (GSK3B1). Recently, studies conducted in Huh7.5 cells using a HCV replicon showed that treatment with a GSK3β inhibitor affected viral replication cycle late during infection, very likely at the assembly and release of viral particles,^8^ ^)^ which was confirmed by the findings in the current study.

Future studies that demonstrate a participation of cellular proteins such as GSK3β in viral infections may allow potential use as a specific therapeutic target for the treatment of infections, capitalising on participation of the kinase in later steps of the signalling pathway. The role of GSK3β in the development of the Dengue disease and the immune-pathogenic mechanisms responsible for severe Dengue fever has been described. Since there is no vaccine or drugs currently available for the treatment of Dengue fever, GSK3B inhibition could counteract or reduce complications from the disease.

Therapeutic use of PI3K/Akt inhibitors has been applied in patients with different types of cancer,^29^ whereas GSK3β protein inhibitors are used in the treatment of neurodegenerative diseases such as Alzheimer’s disease.^30^ The availability of pharmacological inhibitors against proteins involved in this signalling pathway for the treatment of chronic diseases would provide opportunities for rapid evaluation of their potential use in treatments of viral diseases such as Dengue fever. However, given the broad spectrum of metabolic pathways that may be impacted and the regulatory role of these proteins in some essential cellular processes, such as regulation of glucose metabolism described for GSK3β, the identification of possible side effects of these inhibitors would be necessary.

*In conclusions* - In this work, we describe the involvement of the GSK3β during DENV-2 infection of Huh7 and Vero cells, in which the kinase specifically modulates late stages of infection, during possible activation of apoptosis to promote the viral release from infected cells. These findings indicate the potential role of GSK3β during DENV-2 infection process and to some extent, elucidate the complex network of intracellular interactions triggered by the virus in infected cells, aimed at maximising the viral replication process. Although the involvement of the PI3K/Akt signalling pathway in Dengue virus infection has already been described, participation of downstream effectors is very diverse, and little is known about these cellular proteins. Aside well-described roles of GSK3β in process of glucose metabolism and different cellular processes, growing evidence supports its participation in induction of apoptosis in some viral infections such as HIV-1, VZV, HCV, among others. Further studies are required to advance our knowledge and fully describe the participation of cellular proteins such as GSK3β in viral pathogenesis.
